# Targeted axillary dissection reduces residual nodal disease in clinically node- positive breast cancer after neoadjuvant chemotherapy

**DOI:** 10.1186/s12957-024-03413-6

**Published:** 2024-07-06

**Authors:** Neslihan Cabıoğlu, Hasan Karanlık, Ravza Yılmaz, Selman Emiroğlu, Mustafa Tükenmez, Süleyman Bademler, Duygu Has Şimşek, Tarık Recep Kantarcı, İnci Kızıldağ Yirgin, Aysel Bayram, Memduh Dursun

**Affiliations:** 1 Department of Surgery, Istanbul University, Istanbul Faculty of Medicine, Istanbul, Türkiye; 2 Department of Surgical Oncology, Istanbul University, Institute of Oncology, Istanbul, Türkiye; 3 Department of Radiology, Istanbul University, Istanbul Faculty of Medicine, Istanbul, Türkiye; 4 Department of Nuclear Medicine, Istanbul University, Istanbul Faculty of Medicine, Istanbul, Türkiye; 5 Department of Radiology, Istanbul University, Institute of Oncology, Istanbul, Türkiye; 6 Department of Pathology, Istanbul University, Istanbul Faculty of Medicine, Istanbul, Türkiye

**Keywords:** Sentinel lymph node biopsy, Neoadjuvant chemotherapy, Targeted axillary dissection, Clipped node, Wire-localisation, Radio-guided occult lesion localisation (ROLL), CT-guided localisation, Residual nodal disease

## Abstract

**Background:**

Any advantage of performing targeted axillary dissection (TAD) compared to sentinel lymph node (SLN) biopsy (﻿SLNB) is under debate in clinically node-positive (cN+) patients diagnosed with breast cancer. Our objective was to assess the feasibility of the removal of the clipped node (RCN) with TAD or without imaging-guided localisation by SLNB to reduce the residual axillary disease in completion axillary lymph node dissection (cALND) in cN+ breast cancer.

**Methods:**

A combined analysis of two prospective cohorts, including 253 patients who underwent SLNB with/without TAD and with/without ALND following NAC, was performed. Finally, 222 patients (cT1-3N1/ycN0M0) with a clipped lymph node that was radiologically visible were analyzed.

**Results:**

Overall, the clipped node was successfully identified in 246 patients (97.2%) by imaging. Of 222 patients, the clipped lymph nodes were non-SLNs in 44 patients (19.8%). Of patients in cohort B (*n*=129) with TAD, the clipped node was successfully removed by preoperative image-guided localisation, or the clipped lymph node was removed as the SLN as detected on preoperative SPECT-CT. Among patients with ypSLN(+) (*n*=109), no significant difference was found in non-SLN positivity at cALND between patients with TAD and RCN (41.7% vs. 46.9%, *p*=0.581). In the subgroup with TAD with axillary lymph node dissection (ALND; *n*=60), however, patients with a lymph node (LN) ratio (LNR) less than 50% and one metastatic LN in the TAD specimen were found to have significantly decreased non-SLN positivity compared to others (27.6% vs. 54.8%, *p*=0.032, and 22.2% vs. 50%, *p*=0.046).

**Conclusions:**

TAD by imaging-guided localisation is feasible with excellent identification rates of the clipped node. This approach has also been found to reduce the additional non-SLN positivity rate to encourage omitting ALND in patients with a low metastatic burden undergoing TAD.

## Introduction

Axillary nodal status is a major indicator of the clinical prognosis and decision-making criteria for the treatment of breast cancer. Neoadjuvant chemotherapy (NAC) can potentially eradicate axillary metastasis in almost half of patients by minimizing axillary surgery from axillary lymph node dissection (ALND) to sentinel lymph node (SLN) biopsy (SLNB) [1–5]. Clinical trials using the dual method (blue dye and radiotracer) and excising two or more sentinel nodes have reported decreased false-negative rates (FNR) less than 10% [6–9]. Furthermore, some studies have attempted to decrease the FNRs by removing the clipped node either alone or with SLNs to increase the accuracy of SLNB as a technique called targeted axillary dissection (TAD), which improves the FNRs to less than 5% [10–13].

Most studies have utilized either wire-guided localisation (WGL) or I^125^ radioactive seed placement to target the clipped node with high success rates [10, 11, 14–16]. TAD also contains other methods, including charcoal injection into the clipped node before surgery, intraoperative ultrasound use, magnetic seed localisation, or radio-guided occult lesion localisation (ROLL) to guide surgical removal of the marked nodes [17–26]. Localisation of the clipped nodes with wire placement should also be studied under computed tomography (CT)-guidance in addition to US guidance to improve the detection rates of the clips, as validated previously [27, 28].

The primary aim of this study was to evaluate the feasibility of targeted removal of the clipped node using various imaging methods, including wire-guided or radio-guided occult lesion localisation (ROLL) under US or CT, in addition to SLNB, in initially clinically node-positive patients receiving NAC. The secondary aim was to determine the advantage of TAD in decreasing residual lymph node positivity in patients who underwent ALND due to a positive SLNB or clipped node.

### Material and methods

Between June 2017 and October 2022, a prospective study was performed in patients diagnosed with clinically node-positive breast cancer (cT1-3, N1M0/ycN0) to determine the feasibility of TAD using various imaging methods. The study was approved by the Istanbul University Ethics Committee, and informed consent was obtained from all patients. The results were combined with those of a previous prospective study that demonstrated improvement in FNRs with intraoperative identification of clipped nodes in patients undergoing SLNB after NAC [13].

**Fig. 1:**
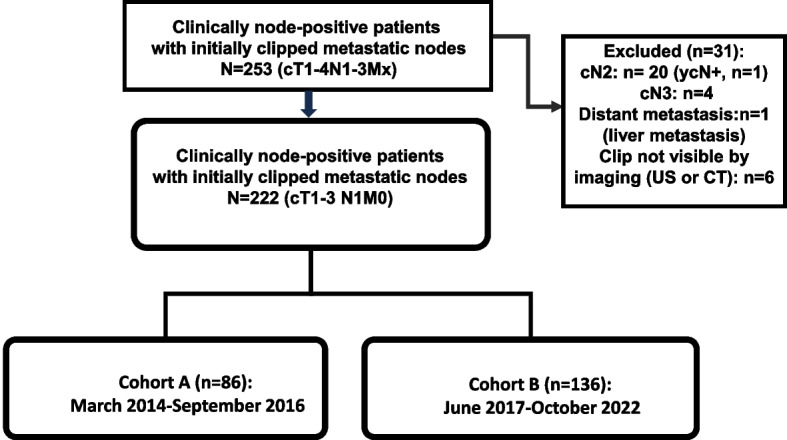
Study Cohort

A sum of 2 prospective cohorts, 253 consecutive patients who underwent surgery between March 2014 and October 2022 were analyzed. Of the 253 patients, the clipped node was not visible in 13 patients (5.1%) on ultrasound. Of these, the clipped node was successfully identified in six patients by CT. Finally, the clipped node could not be found in seven cases (2.8%) by any imaging in the current study, and axillary dissection was performed for those cases. Therefore, the clipped node was successfully identified in 246 patients (97.2%) by using US or CT. Overall (*n*=253), the mapping success rate of SLNB was 92.9%, except for 18 patients with mapping failure. Among patients with mapping failure in whom the SLN could not be identified, two underwent ALND because of suspicious or positive intraoperative evaluation of the clipped nodes. Patients with distant metastases or clinical T4, N2, or N3 disease or any suspicious nodes on US in preoperative evaluation following NAC, and 6 patients with invisible clipped nodes on imaging were excluded from the final analysis (Fig. 1). A total of 222 patients (cT1-3N1/ycN0M0) with clinically node-negative disease determined by physical examination and imaging following NAC and with a clipped lymph node that was radiologically visible (US or CT) were analyzed, including 85 patients in cohort A and 137 patients in cohort B.

### Pretreatment axillary nodal evaluation and clip placement procedure

All patients with clinical initially node-positive breast cancer with suspicious ipsilateral axillary lymph nodes underwent routine breast imaging, including breast ultrasound (US), mammography, magnetic resonance imaging (MRI), and ^18^F-fluorodeoxyglucose positron emission tomography/computed tomography (^18^F-FDG-PET/CT) at our institution.

Axillary US and all interventional examinations under US guidance were performed with either a 7–12-MHz linear array transducer (LOGIQ 9) from GE Healthcare (Milwaukee, WI), or a linear (i18LX5) transducer: an Aplio i800 scanner (Canon Medical Systems, Tustin, CA, USA). The index node was identified using one or more of the following criteria, as previously described [13, 29–31]. Before starting NAC, commercially available titanium clips (UltraClip Dual Trigger Breast Tissue Marker, 17G × 10 cm Needle – Ribbon., C. R. Bard, Inc., NJ, USA) were used to mark the biopsy-proven metastatic axillary lymph node(s) under ultrasound guidance.

### Systemic treatment

The majority of patients (*n*=191, 86%) received four cycles of AC (adriamycin, 60mg/m^2^ and cyclophosphamide, 500 mg/m^2^) plus 12 cycles of weekly paclitaxel (80 mg/m^2^). Of these, three patients (1.3%) with triple-negative disease also received carboplatin following weekly paclitaxel with/without immunotherapy. Of the remaining patients, 26 (11.7%) had four cycles of AC plus 4 cycles of docetaxel (75 mg/m^2^) and 2 (1%) had docetaxel with cyclophosphamide. All patients with HER2-neu positive disease (*n*=79, 35.6%) additionally received trastuzumab therapy (2 mg/kg) with or without pertuzumab in addition to taxanes.

### Axillary nodal evaluation following NAC and marking before targeted axillary surgery

**Fig. 2:**
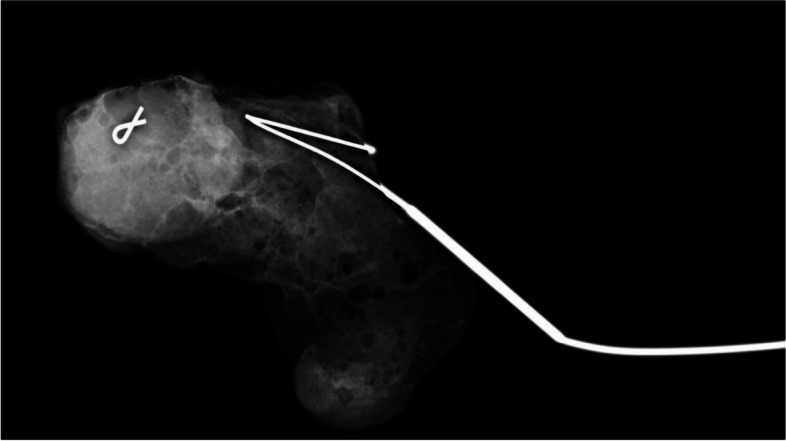
Specimen radiograph of a SLN removed by wire localisation with a clip inside

**Fig. 3:**
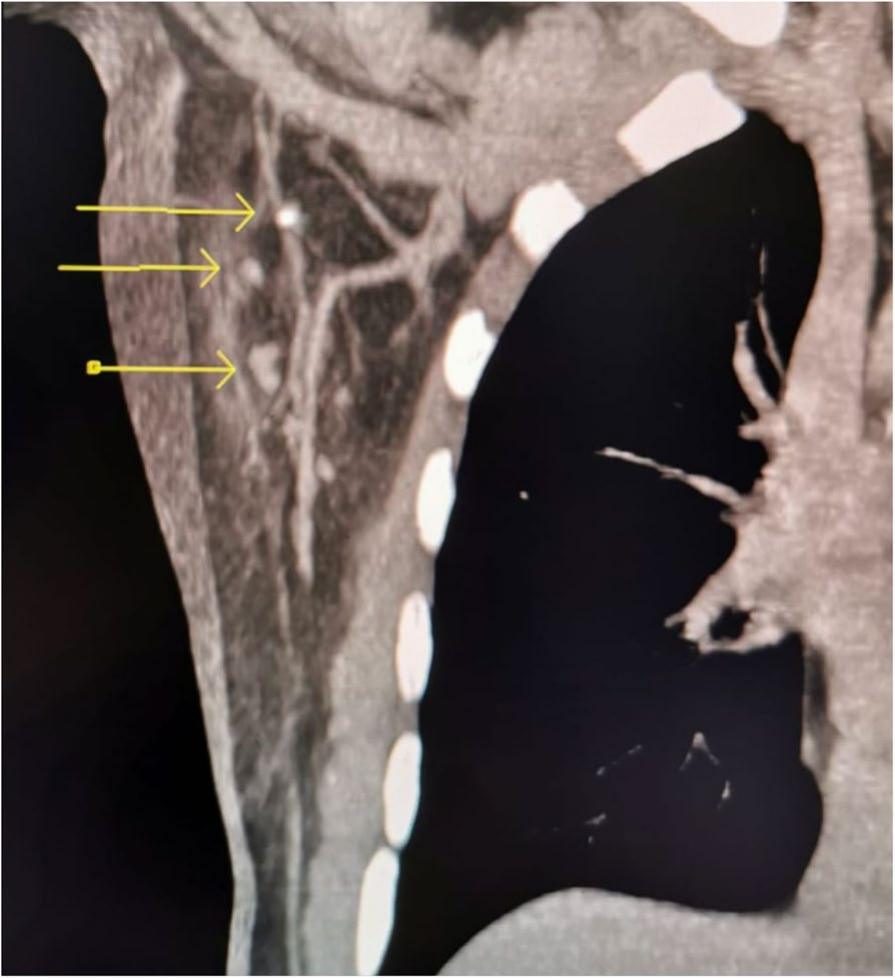
The upper arrow shows the marked lymph node with a clip inside, whereas the lower arrows indicate the other lymph nodes visible under computed tomography. The clipped lymph node was removed with ROLL along with the suspicious lymph node indicated with the lower arrow below the other lymph nodes

In all patients, the chemotherapy response was monitored using both breast MRI and focused axillary US following the completion of NAC. If the clipped lymph node could not be seen on US after NAC, evaluating pre-NAC and post-NAC MRI findings in comparison to the ultrasound findings might help to determine the clipped lymph node in such cases. Otherwise, the clipped node was marked under CT guidance [27, 28]. Based on the surgeon and radiologist's preference, the clipped lymph node was localized with radioactive ^99m^Tc- macroaggregated albumin or wire (WGL) under US or CT guidance on the day of surgery (Figs. 2 and 3).

### ROLL procedure

Approximately 0.3 mCi ^99m^Tc- macroaggregated albumin was injected into the clipped and/or suspicious lymph nodes percutaneously with the guidance of ultrasonography or CT. A dedicated gamma probe (Europrobe II, USA) was intraoperatively used to remove the labeled lymph nodes.

## Targeted axillary dissection and pathological evaluation

SLNB was performed with only blue dye in 129 patients (58.1%), and with the combined blue dye and ^99^Tc-nanocolloid injection (0.3-0.5 mCi) technique in 93 patients (41.9%). Palpable suspicious lymph nodes were also considered SLNs, as described previously [32], and were sent for intraoperative pathological evaluation. In cohort A, no imaging guided-localisation was necessary for the removal of the clipped node (RCN) in addition to the SLNB. However, TAD was defined as SLNB along with the removal of the clipped node with the imaging-guided localisation techniques. The marked clipped lymph nodes were removed using ROLL or WGL or carbon dye marking. Patients who underwent TAD by ROLL underwent SLNB using blue dye only to prevent interference of the same signal from the radioisotopes. Specimen radiography was performed to confirm whether the clipped lymph nodes were removed in both cohort A and B.

Intraoperative lymph node evaluation was performed by imprint cytology. SLNs along with the clipped node were examined for final definitive pathology, as described previously [33]. The final pathology has also described the lymph node response to NAC as the presence of regression with/without metastatic involvement. Pathological complete response (pCR) was defined as the absence of invasive cancer in the breast and axillary lymph nodes [34]. The AJCC Staging 8th edition has been used in the clinical and pathological TNM classification [35]. The tumor subtypes according to IHC staining were analyzed using Ki-67 <20% as the low cut-off value, as described previously [36].

### Statistical analysis

The software program SPSS 26 (Statistical Package for Social Sciences; IBM Corp., Armonk, NY, USA) was used for statistical analyses. The associations between the categorical variables were determined using Fisher’s exact test or the continuity correction test (Pearson Chi-Square) in a two-tailed univariate analysis. The differences between nonparametric continuous variables were estimated using the Mann-Whitney U test. The lymph node ratio (LNR) was calculated as the number of metastatic lymph nodes divided by the total number of lymph nodes removed. Statistical significance was set at *p* ≤0.05.

## Results

### Patient characteristics

The demographic features and surgical and clinicopathological characteristics of the 222 patients are shown in Table 1. The median age of the patients was 45 (24-73). All patients had cN1 tumors, whereas the majority (*n*=143, 64.4%) had cT2 tumors. The median number of SLNs (range, min-max, IQR) was 3 (1-8, IQR:2-4), respectively. Clipped lymph nodes were detected in 178 patients (80.2%) with SLNs and 44 patients (19.8%) with non-SLNs.

**Table 1:** Clinical and pathological characteristics of patients presented with cN1 who had sentinel lymph node biopsy (SLNB) with removal of the clipped node (RCN) without localisation techniques compared to those with targeted axillary dissection (TAD) by localisation techniques following neoadjuvant chemotherapy

**Patient Characteristics(*N*=222)**	**All** **(*n*=222)**	**TAD** **(*n*=129)**	**RCN by SLNB** **(*n*=93)**	***p*-Value**
**Age, median(range)**	45(24-73)	46(24-73)	45(28-66)	*0.214*^*a*^
Clinical* Tumor Stage before Neoadjuvant Chemotherapy, n(%)				*0.074*^*b*^
T1	33(14.9)	21(16.3)	12(12.9)	
T2	143(64.4)	89(69)	54(58.1)	
T3	33(14.9)	13(10.1)	20(21.5)	
T4	13(5.9)	6(4.7)	7(7.5)	
**Breast Surgery, n(%)**				*0.445*^*b*^
Breast Conserving Surgery	126(56.8)	76(58.9)	50(53.8)	
Mastectomy	96(43.2)	53(41.1)	43(46.2)	
**Histopathology, n(%)**				*0.136*^*b*^
Invasive ductal cancer	201(90.5)	121(93.8)	80(86)	
Invasive lobular cancer	10(4.5)	5(3.9)	5(5.4)	
Invasive ductal/lobular type	6(2.7)	1(0.8)	5(5.4)	
Other	5(2.3)	2(1.6)	3(3.2)	
**IHC-based subtype, n(%)**				*0.452*^*b*^
Luminal A	31(14)	18(14)	13(14)	
Luminal B	77(34.7)	42(32.6)	35(37.6)	
Luminal- HER2-neu	47(21.2)	24(18.6)	23(24.7)	
Non-luminal HER2-neu	32(14.4)	21(16.3)	11(11.8)	
Triple negative breast cancer	35(15.8)	24(18.6)	11(11.8)	
**Treatment response, n(%)**				
Pathologic Complete Response	62(27.9)	37(28.7)	25(26.9)	*0.768*^*b*^
Breast Pathologic Complete Response	84(37.8)	53(41.1)	31(33.3)	*0.240*^*b*^
Axillary Pathologic Complete Response	91(41)	52(40.3)	39(41.9)	*0.808*^*b*^

^a^Mann-Whitney U test

^b^Pearson’s chi-square test

Of the patients in cohort B (*n*=129) with TAD, the clipped node was successfully removed by WGL in 75 patients (63.6%) and by ROLL in nine patients (7.6%), carbon dye marking in nine patients (*n*=9, 7.6%), or skin marking (*n*=25, 21.2%) by preoperative localisation with US (*n*=112) or CT-guidance (*n*=6). In the remaining 11 patients (8.1%), the clipped lymph node was removed as the SLN that was detected and localized on preoperative SPECT-CT. Furthermore, the clipped node was detected as the SLN in the specimen graph of eight patients.

### Surgical and pathological features

Of the patients in Cohort A (*n*=85), the majority (*n*=76, 89.4%) underwent ALND regardless of the SLN pathology due to the study protocol to estimate the false negative rate to assess the feasibility of clipping the metastatic lymph node versus SLNB alone. Of the patients in cohort B (*n*=137), the majority (*n*=50, 89.3%) with ypN0 (*n*=56) underwent SLNB with removal of the clipped lymph node. The remaining six patients underwent ALND according to the surgeon’s preference due to suspicious palpable lymph nodes.

Of patients (*n*=81) with ypN(+) disease in cohort B, 62 patients (76.5%) underwent axillary dissection, whereas 19 patients underwent only SLNB due to a negative intraoperative pathological evaluation and/or limited metastatic nodal involvement in the definitive pathology. These patients were discussed on tumor board, ALND was omitted because of the limited low-volume metastatic disease in the lymph nodes, and patients underwent level 1-3 axillary radiation therapy in addition to the chest wall region. Overall, the final definitive pathology of the clipped lymph node showed regression in 72 patients (32.5%), metastatic involvement with/without regression in 70 (31.5%) and 50 (22.5%) patients, respectively, and reactive changes in 30 (13.5%) patients. Of 44 patients with a clipped node as a non-SLN, 15 had ypN0-disease, whereas 29 had ypN+ disease. Of those, only eight patients had breast pCR, and six of them had metastases in the clipped node. Of the six patients with a metastatic clipped node detected as non-SLN, only 3 of them had non-luminal pathology. Therefore, identifying the metastatic lymph nodes in the clipped node among 222 patients altered the systemic treatment in only two patients (0.9%) with ypN+ triple-negative breast cancer and HER2-positive disease who received Xeloda (*n*=1) or TDM-1 (*n*=1) as adjuvant treatment, respectively.

### Comparison of patients with TAD versus RCN without localisation

Patients in cohort A and B with RCN (*n*=93) were compared to those in cohort B, who underwent TAD (*n*=129) in terms of clinical and pathological characteristics and SLNB features. No significant differences were found in the median age, clinical T and N stage, breast surgery type, pathologic complete response (pCR), breast pCR, axillary and breast pCR, and breast pathology subtype based on H&E and immunohistochemistry staining (Table 1). However, patients with TAD were more likely to undergo SLNB using the combined technique (51.9% vs. 28%, *p*<0.00). Furthermore, the median SLN number (IQR) was found to be significantly increased in the cohort with TAD (3; 2-4) versus those with RCN (2; 1-3), and patients with TAD were more likely to have SLNs ≥3 removed than those other (62.5% vs. 34.4%, *p*<0.001; Table 2).

**Table 2:** Axillary surgery and sentinel lymph node biopsy (SLNB) characteristics of patients presented with cN1 who had SLNB with the removal of the clipped node removal (RCN) with (TAD)/without localisation techniques after neoadjuvant chemotherapy

**SLNB Characteristics**(***N***=222)	**All** **(*n*=222)**	**TAD** **(*n*=129)**	**RCN by SLNB** **(*n*=93)**	***p*-Value**
**SLNB Method, n(%)**				***<0.001***^***a***^
Blue dye only (isosulphane blue)	129(58.1)	62(48.1)	67(72)	
Combined technique	93(41.9)	67(51.9)	26(28)	
**SLNB number, median (IQR)(n=222)**	3(2-4)	3(2-4)	2(1-3)	***<0.001***^***b***^
1 SLN	48(21.6)	18(14.1)	29(31.2)	***<0.001***^***a***^
2 SLN	62(27.9)	30(23.4)	32(34.4)	
≥3 SLN	112(50.5)	80(62.5)	32(34.4)	
**Targeted Axillary Dissection Procedure, n(%)**				*NA*
ROLL	9(7)	9(7)	NA	
Wire	75(58.1)	75(58.1)	NA	
Carbon	9(7)	9(7)	NA	
SPECT/CT	11(8.1)	11(8.1)	NA	
Ultrasound-guided skin localization	25(19.4)	25(19.4)	NA	

^a^Pearson’s chi-square test

^b^Mann-Whitney U test

Not applicable

Among those who underwent completion ALND, no significant difference was found in the non-sentinel lymph node positivity (non-SLNBP) between patients with TAD and RCN by removal of either the SLNs alone, the clipped node alone, or both (Table 3). However, among those with a metastatic lymph node (LN) in the removed LN specimen, patients with TAD were less likely to have non-sentinel lymph node positivity compared to those with the removal of both the clipped and SLNs without a localisation technique that did not reach statistical significance (27.6% vs. 50%, *p*=0.074). Among those with TAD and ALND (*n*=60), patients with one metastatic LN and an LNR of less than 50% in the TAD specimen were found to have significantly decreased non-SLNB positivity (27.6% vs. 54.8%, *p*=0.032, and 22.2% vs. 50%, *p*=0.046, Table 4).

**Table 3:** The non-sentinel lymph node positivity (non-SLNP) rates in the completion axillary node dissection (ALND) in ypN (+) patients who underwent axillary lymph node dissection (*n*=109)

**Non-SLNP rates according to the axillary lymph node characteristics**	**All(%)**	**TAD (*n*=60)**	**RCN (*n*=49)**	***p*-Value**
By removal of the clipped node with SLNs	44% (48/109)	41.7% (25/60)	46.9% (23/49)	*0.581*
By removal of SLNs alone	51.4% (56/109)	51.7% (31/60)	51% (25/49)	*0.946*
By removal of the clipped lymph node alone	70.6% (77/109)	66.7% (40/60)	75.5% (37/49)	*0.313*

*TAD* Targeted axillary dissection with localisation techniques, *RCN* Removal of the clipped node by SLNB without localisation techniques

Pearson Chi-square test was used in the analyses

**Table 4:** Non-sentinel lymph node (SLN) positivity rates according to lymph node (LN) characteristics among patients who underwent targeted axillary dissection (*n*=60)

**LN Characteristics**	**Non-SLN positivity (%)**	***p*-value**
**Number of metastatic LNs**		*0.032*
1 metastatic LN	27.6% (8/29)	
>1 metastatic LN	54.8% (17/31)	
**Lymph node ratio (%)**		*0.046*
<50%	22.2% (4/18)	
≥50%	50%(21/42)	

*P-values* were calculated with Pearson Chi-square test

## Discussion

TAD has recently become popular for axillary staging after NAC, with the rationale of causing less morbidity than ALND and decreasing FNRs [9–13, 23, 24, 37, 38]. The Sen-Ta prospective registry trial from 50 centers in Germany reported that the clipped node could be successfully excised in 329 of 423 patients (77.8%) who underwent NAC due to clinically node-positive disease. The FNR for targeted LN biopsy (TLNB) was 7.2%, whereas 4.3% FNR was reported for TAD including SLN in addition to TLNB [23]. A recent meta-analysis found a similar FNR as a pooled analysis of nine studies, including 366 patients as 6.28% for TLNB and 5.18% of 13 studies with 521 patients with TAD, with an overall success rate of 90% to retrieve the clipped node [24].

Targeted axillary surgery procedures after NAC included removal of the marked LN as TLNB) by different techniques, including I^125^ radioactive seed or wire or magnetic seed placement techniques before the surgery or a combination of SLNB and TLNB as TAD [11, 14–24, 37, 38]. However, there are some safety concerns regarding the radioactivity of iodine seeds in many European countries and the USA, although the dose of iodine seeds is low. Hellingman et al. recently evaluated whether ROLL of clip-marked proven tumor-positive lymph nodes was feasible in patients with breast cancer in clinical practice [39]. After NAC, ^99m^Tc- macroaggregated albumin (ROLL) was injected into the clip-marked lymph nodes (*n*=38) of 37 patients. The clip was visible on ultrasound in 36 procedures (95%), and the clipped node was successfully detected in 33 procedures (87%). Similar to this study, the clipped node was successfully localized with ROLL by US or CT in all cases (*n*=9) in the present study. Blue dye injection alone was used as the SLNB technique in these patients. Removal of the ROLL-marked LN was the only LN pathologically evaluated in one patient due to unsuccessful mapping by blue dye alone. The clipped node was identified with 100% success rate in all cases. All of these studies demonstrate that the utilization of the ROLL procedure to localize and identify clip-marked lymph nodes is feasible.

Other alternative localisation techniques, including wire placement, charcoal, and magnetic seed placement, are becoming more popular in Europe [14–16, 18, 20]﻿. To retrieve the clipped node, wire localisation of the node was performed in the majority of patients (*n*=75) under ultrasound guidance as the TAD technique in the present study, which is one of the largest studies published to date [14]. In concordance with previous studies  [24], we reported a 94.9% success rate in detecting the clipped node by ultrasound, which might be due to our experienced breast radiology team. In the present study, almost 5.1% of the clipped nodes (*n*=13) were not visible in US. Hartmann et al., however, reported that the clipped node identification rate was 70% in 30 patients using the ultrasound-guided wire-placement technique, and the clipped node could not be confirmed by intraoperative radiography in 30% of cases [15]. Therefore, US-guided wire placement was not feasible for clinical use in their series because of the limitations in clip visibility.

CT-guided wire localisation has been reported in five cases with a clinically positive axilla and clipped node before NAC as an alternative technique where the clipped node could not be seen under US  [30]. In our series, six of 13 cases successfully underwent CT-guided wire localisation because the clipped node could not be visualized by US. This strategy increased the final identification rate of the clipped node from 94.9% to 97.2% by using any radiological approach. Therefore, we can conclude that wire-localisation under US or CT guidance was feasible at our institution with a high identification rate of clipped nodes in our series.

The added advantage of clipping the metastatic lymph nodes with or without using localisation techniques has been a debate in the recent literature compared to the standard SLNB techniques, either with a dual tracer or a single agent. Of note, the clipped node was found to be a non-SLN in almost 20% of patients in our study, which is a lower rate than the previous series [11], which might be due to the high number of SLNs (≥3) removed in concordance with the recent reports by Montagna et al. and Weiss et al. [38, 39]. In the series of Montagna et al., clipped nodes were reported in 12% (31/251) of patients as non-SLNs, with a median number of SLN of 4 [40]. Moreover, no axillary recurrence was observed in 18 patients who underwent SLNB only at a median follow-up of 55 months, in whom the clipped node could not be retrieved. Furthermore, Weiss et al. demonstrated the clipped LN as a non-SLN in 19% of cN1 patients, and the pathology of the clipped node did not change the systemic adjuvant therapy, similar to our findings in the present study [41].

The majority of the literature regarding SLNB and TAD after NAC is based on mapping techniques using dual tracers such as injection of radioisotope and blue dye to improve the FNRs to <10% [6–9, 11, 12]. However, we previously published our experience with TAD reporting an acceptable FNR of 10.5% with a SLNB by blue dye-only, whereas removal of at least two SLNs further reduced the FNR to less than 5% regardless of the SLNB technique among patients with cT3N1 [13]. Furthermore, we recently demonstrated the oncological safety of SLNB without ALND in selected cN (+) patients with breast and/or nodal pCR or low-volume residual nodal disease after NAC. The majority of these patients underwent SLNB with blue dye only in this multicentric trial [42].

In patients with a positive SLN after NAC, the likelihood of non-SLN positivity during ALND has been reported to be higher than 50% [43–45]. Leonardi et al. demonstrated that the number of positive SLNs, higher ratio of positive SLNs/total SLNs, larger SLN metastasis size, SLN extracapsular extension, and aggressive tumor biology (HER2+ vs. HER2-) remained significant predictors of additional lymph node metastasis in ALND [45]. In our series of patients with cT3N1 (*n*=109), the additional non-SLN positivity rates at the completion of ALND in patients with an intraoperative pathological positive node were found to be 51.4% with the SLNB technique alone, 70.6% by removal of the clipped lymph node alone, and 44% by using both techniques. In concordance with some studies [45], we also found a lower likelihood of non-SLN positivity rate at the completion of ALND of less than 28% in a subgroup of patients with a low axillary metastatic burden at the TAD. However, further studies with larger sample sizes should be performed to confirm these findings.

In conclusion, our findings suggest that removal of the clipped lymph node under the guidance of various radiological methods, including wire or ROLL, is feasible with a high identification rate of the clipped node. The residual axillary disease is minimal if both the sentinel lymph nodes and the clipped nodes are removed at the axillary surgery. Retrieval of the clipped node in addition to SLNB did not change the adjuvant treatment in patients but reduced the non-SLN positivity rates even more compared to each technique alone in cALND, especially in those with low-volume metastatic disease. However, the clinical significance of this finding remains to be proven in ongoing prospective studies to determine the oncological safety of omitting ALND in selected ypN(+) patients, including those with a low metastatic burden undergoing TAD [46–50].

## Data Availability

Availability of data and materials: The datasets generated and analyzed in the present study are not publicly available due to privacy, but can be obtained from the corresponding author on reasonable request.
